# Pattern and determinants of hospitalization during heat waves: an ecologic study

**DOI:** 10.1186/1471-2458-7-200

**Published:** 2007-08-09

**Authors:** Giuseppe Mastrangelo, Ugo Fedeli, Cristiana Visentin, Giovanni Milan, Emanuela Fadda, Paolo Spolaore

**Affiliations:** 1 Department of Environmental Medicine and Public Health, University of Padova, Padova, Italy; 2 SER – Epidemiological Department, Veneto Region, Castelfranco Veneto, Italy

## Abstract

**Background:**

Numerous studies have investigated mortality during a heatwave, while few have quantified heat associated morbidity. Our aim was to investigate the relationship between hospital admissions and intensity, duration and timing of heatwave across the summer months.

**Methods:**

The study area (Veneto Region, Italy) holds 4577408 inhabitants (on January 1^st^, 2003), and is subdivided in seven provinces with 60 hospitals and about 20000 beds for acute care. Five consecutive heatwaves (three or more consecutive days with Humidex above 40°C) occurred during summer 2002 and 2003 in the region. From the regional computerized archive of hospital discharge records, we extracted the daily count of hospital admissions for people aged ≥75, from June 1 through August 31 in 2002 and 2003. Among people aged over 74 years, daily hospital admissions for disorders of fluid and electrolyte balance, acute renal failure, and heat stroke (grouped in a single nosologic entity, heat diseases, HD), respiratory diseases (RD), circulatory diseases (CD), and a reference category chosen a priori (fractures of the femur, FF) were independently analyzed by Generalized Estimating Equations.

**Results:**

Heatwave duration, not intensity, increased the risk of hospital admissions for HD and RD by, respectively, 16% (p < .0001) and 5% (p < .0001) with each additional day of heatwave duration. At least four consecutive hot humid days were required to observe a major increase in hospital admissions, the excesses being more than twofold for HD (p < .0001) and about 50% for RD (p < .0001). Hospital admissions for HD peaked equally at the first heatwave (early June) and last heatwave (August) in 2004 as did RD. No correlation was found for FF or CD admissions.

**Conclusion:**

The first four days of an heatwave had only minor effects, thus supporting heat health systems where alerts are based on duration of hot humid days. Although the finding is based on a single late summer heatwave, adaptations to extreme temperature in late summer seem to be unlikely.

## Background

Numerous studies have investigated mortality during a heatwave, while there have been comparatively few studies that have quantified heat associated morbidity. Only a single paper examined the whole burden of heat-related illness by analyzing hospital discharge diagnoses according to International Classification of Diseases, 9th Revision (ICD-9): during the 1995 heatwave in Chicago there were 35% more than expected admissions among patients aged >65 years, mainly due to dehydratation, heat stroke, heat exhaustion and acute renal failure [1]. While a study carried out in greater London from 1994 to 2000 found a small or absent impact of heat on admissions among the elderly [2], a recent paper reported an excess of emergency hospital admissions during the 2003 heatwave in England among those aged 75 or more [3].

In examining the effect of heatwaves, the timing of these episodes across the summer months must be considered. It has been reported that heat waves occurring in the spring or early summer often resulted in more deaths than heatwaves occurring later in the summer [4-7]. Similarly, the first heatwave in a year with multiple events could generate higher rates of hospital admission than subsequent heatwaves, even if they are longer or more intense, because individuals may have acquired physiologic adaptations or have implemented behavioural changes following their initial exposure to high ambient temperature. This has never been reported, however.

A study found a significantly increased mortality with heatwave duration and timing within the summer season, but not with intensity of hot weather [8]. A similar relationship has never been investigated for hospital admissions. It should be examined whether a minimum duration of heatwaves is required to produce a health impact.

This would suggest that individual hot days are less significant than consecutive hot days, in which individuals do not receive sufficient respite from the heat to recover physiologically.

In this report, we examine all of these issues. In order to show the relationship between hospitalization pattern and, on the other hand, intensity duration or timing of hot spells, we compare five consecutive heatwaves (one occurred in 2002 and four in 2003) and the associated hospital admissions in people aged 75 and more years, residents in the Veneto Region, North-Eastern part of Italy.

## Methods

The study area holds 4577408 inhabitants (on January 1^st^, 2003), and is subdivided in seven provinces with 60 hospitals and about 20000 beds for acute care. From the regional computerized archive of hospital discharge records, we extracted the daily count of hospital admissions for people aged ≥75, from June 1 through August 31 in 2002 and 2003. A satisfactory level of accuracy of primary discharge diagnoses provided by this archive has been verified for circulatory disease [9,10]. We analyzed the primary discharge diagnosis considering the following nosologic groups: Infectious diseases (ICD-9 codes 001–139); Tumors (140–239); Endocrine, Nutritional, and Metabolic Diseases and Immunity Disorders (240–279); Diseases of Blood and Blood Forming Organs (280–289); Mental Disorders (290–319); Diseases of Nervous System and Sense Organs (320–389); Diseases of Circulatory System (390–459); Diseases of Respiratory System (460–519); Diseases of Digestive System (520–579); Diseases of Genitourinary System (580–629); Diseases of Skin and Subcutaneous Tissue (680–709); Diseases of Musculoskeletal and Connective Tissue (710–739); Signs, Symptoms and Ill-Defined Conditions (780–799); Injury and Poisoning (800–999). Furthermore, we generated a new nosologic entity (Heat Diseases) by grouping those discharge diagnoses found to be heat-related by Semenza [1]: Disorders of fluid and electrolyte balance (276); Acute Renal Failure (584); and Heat Stroke (992). Lastly, Fractures of Femur (820–821) were chosen a priori as a reference diagnostic category not related to heat.

Ethical approval was not required as the evaluation of the health impact of heatwaves is a mandatory activity of the Epidemiological Department of the Veneto Region according to a regional decree (n. 2067/2006). All hospital discharge records were anonymized and handled according to national and regional rules on confidentiality.

Humidex is a measure of perceived heat that results from the combined effect of excessive humidity and high temperature. The Weather Service of Environment Canada [11] uses Humidex numbers to inform the public when conditions of heat and humidity are: comfortable (20–29°C); some discomfort (30–39°C); great discomfort (40–45°C); dangerous (above 45°C); heat stroke imminent (above 54°C).

We obtained from the seven weather stations located in each provincial capital of our region the daily values of maximum temperature (°C) and humidity occurring at the same hour, from which we calculated Humidex according to Masterton and Richardson [12]. Since the spatial variation of the bioclimatic indices was quite low, the average value was considered representative for the entire region [13]. We adopted the usual definition of heatwave as three or more consecutive days above a threshold [14], setting the latter at a value of Humidex equal to 40°C, a value classified as dangerous in an Italian study of heat-related mortality [15].

Epidemiological evaluation of the data was undertaken using both simple analysis and multivariate models. Analysis was carried out for the entire period of the study, subsuming 184 days, 92 for summer 2002 and 92 for summer 2003. Ours is therefore an ecologic study of time relation between hospital admissions and heat, day being the standard statistical unit. In the univariate analysis, scatter diagram was produced showing the values for each cause of hospitalization and heatwave intensity, duration, and timing within summer. Data included outcome variables (daily count of hospital admission by cause), daily value of heatwave intensity and duration and day of week in order to account for the weekly cyclical pattern of admissions (usually lower on weekends). In the multivariate analysis, the parameters of each one of the above variables were estimated through Generalized Estimating Equations (GEE). Modeling the relation of hot weather with hospital admissions was performed using the statistical package STATA 8 [16] through the command "xtgee" with a log link and a Poisson error distribution. Daily counts of hospital admissions within each summer were assumed to be correlated; the correlation structure was modeled using a first order autoregressive structure, which assumes that responses further apart in time will be less correlated than those closer in time. In order to disentangle the effect of heatwave duration from that of intensity, we built a GEE model that included three variables:

• "duration", which was 1 (1^st ^day) through n (last day) within a heatwave, and 0 otherwise;

• "intensity", which was equal to the daily Humidex value within a heatwave, and 0 otherwise;

• and a dummy variable coded as 1 or 0, for days outside or inside a heatwave, respectively.

This parameterization produces estimates of dose-response relationships (duration, or intensity, versus daily count of admissions) limited to the heatwave period, by allowing the response of other days to have a separate estimate [17].

In order to disentangle the effect of heatwave duration from that of timing within summer period, we built another GEE model that included nine dichotomous variables (setting as reference all days outside heatwaves) to take into account five consecutive heatwaves:

• heatwave 1-2002, June 16 through 24, 2002: we separately coded the first (4 days) and second part (5 days) of the heatwave;

• heatwave 1-2003, June 10 through 16, 2003: we separately coded the first (4 days) and second part (3 days);

• heatwave 2-2003, June 23 through 26, 2003 (4 days)

• heatwave 3-2003, July 21 through 23, 2003, (3 days)

• heatwave 4-2003, August 3 through 14, 2003; we separately coded the first (4 days), the second (4 days) and the last part (4 days).

## Results

In the whole observation period, the mean (standard deviation) of daily Humidex values was 36.5 (4.2) °C. Means (standard deviations) of daily hospital admissions were: 5.7 (4.3) for Heat Diseases (HD); 39.7 (11.5) for Respiratory Diseases (RD); 124.6 (29.1) for Diseases of the Circulatory System (CD) and 11.7 (3.4) for Fractures of Femur (FF).

**Figure 1:**
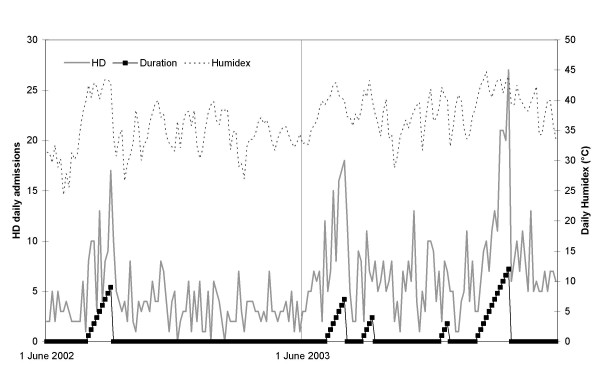
Heatwave duration, daily admissions for heat disease (HD) and daily Humidex, 1 June–31 August 2002 and 2003.

**Figure 2:**
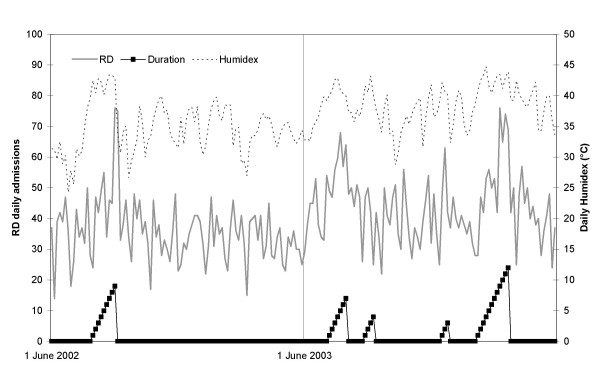
Heatwave duration, daily admissions for respiratory disease (RD) and daily Humidex, 1 June–31 August 2002 and 2003.

The daily values of heatwave duration, humidex and admissions for HD or RD are shown in Figure 1 and 2, respectively. It can be seen that both diseases seem more correlated to heatwave duration than intensity; and that the last heatwave, which was the longest and most intense among multiple events of 2003, generated higher rates of hospital admission than the first one, stressing the fact that subjects did not acquire physiologic adaptations following their initial exposure to high ambient temperature.

If Disorders of Fluid and Electrolyte Balance (276) and Acute Renal Failure (584) were excluded from the corresponding Main Groups, the latter (Endocrine Nutritional Metabolic Diseases and Immunity Disorders, and Diseases of Genitourinary System) as well as all other nosologic groups and FF did not show any relationship with heatwave characteristics, using information obtained in the graphical representations (data not shown).

Table 1 shows that heatwave duration, not intensity, increased the risk of hospital admissions for HD and RD. The excess risk (adjusted for weekly periodicity) rose by 16% (p < .0001) and 5% (p < .0001) for HD and RD, respectively, with each more day of heatwave duration. By contrast, FF and CD did not show any increase with duration or intensity.

**Table 1:** Incidence rate ratio (IRR), 95% confidence interval (95%CI), and p-value estimated by Generalized Estimating Equations.

	HD		RD		FF		CD	
	IRR ^a ^(95%CI)	p-value	IRR ^a ^(95%CI)	p-value	IRR ^a ^(95%CI)	p-value	IRR ^a ^(95%CI)	p-value

Humidex within heatwaves (°C)	0.96 (0.88 – 1.04)	0.329	1.00 (0.96 – 1.04)	0.929	0.99 (0.92 – 1.07)	0.750	0.98 (0.95 – 1.00)	0.049
Duration of a heatwave (days)	1.16 (1.12 – 1.20)	0.000	1.05 (1.03 – 1.07)	0.000	0.99 (0.96 – 1.03)	0.563	1.00 (0.99 – 1.01)	0.470
Dummy variable (see text)	0.17 (0.01 – 5.75)	0.327	1.03 (0.23 – 4.72)	0.969	0.53 (0.02 – 13.1)	0.700	0.38 (0.14 – 0.98)	0.045

HD = heat disease; RD = respiratory disease; FF = fractures of femur; CD = circulatory disease

^a ^adjusted for day of week

Heatwaves 1-2002, 1-2003, and 4-2003 greatly influenced hospital admissions for HD and RD in their last part, while in their first part (four days) they had only a minor effect, like did heatwaves 2-2003 and 3-2003 which persisted for 4 and 3 days, respectively. Duration was thus confirmed as major determinant of hospitalization. Furthermore, early occurrence of heatwave did not result in higher hospital admissions than that of the late event within the summer 2003. Again, FF and CD did not show any consistent pattern of correlation (Table 2).

**Table 2:** Incidence rate ratio (IRR), 95% confidence interval (95%CI), and p-value estimated by Generalized Estimating Equations

	HD		RD		FF		CD	
	IRR ^a ^(95%CI)	p-value	IRR ^a ^(95%CI)	p-value	IRR ^a ^(95%CI)	p-value	IRR ^a ^(95%CI)	p-value

Days outside heatwaves	Reference		Reference		Reference		Reference	

Heatwave 1-2002, 1^st ^part	1.65 (1.08 – 2.50)	0.019	1.07 (0.89 – 1.29)	0.441	1.13 (0.84 – 1.52)	0.410	1.03 (0.94 – 1.12)	0.593
Heatwave 1-2002, 2^nd ^part	2.35 (1.70 – 3.25)	0.000	1.37 (1.18 – 1.59)	0.000	1.01 (0.77 – 1.33)	0.951	1.07 (0.99 – 1.17)	0.090
Heatwave 1-2003, 1^st ^part	1.56 (1.04 – 2.36)	0.033	1.30 (1.11 – 1.53)	0.001	1.14 (0.85 – 1.52)	0.384	1.06 (0.97 – 1.16)	0.187
Heatwave 1-2003, 2^nd ^part	3.78 (2.70 – 5.30)	0.000	1.76 (1.49 – 2.07)	0.000	0.87 (0.60 – 1.27)	0.472	1.17 (1.05 – 1.30)	0.003
Heatwave 2-2003	1.17 (0.73 – 1.89)	0.510	1.00 (0.84 – 1.20)	0.993	1.17 (0.88 – 1.55)	0.278	1.01 (0.92 – 1.10)	0.879
Heatwave 3-2003	1.04 (0.60 – 1.83)	0.889	1.22 (1.01 – 1.46)	0.036	0.95 (0.67 – 1.36)	0.788	1.04 (0.94 – 1.14)	0.469
Heatwave 4-2003, 1^st ^part	1.57 (1.02 – 2.41)	0.038	1.14 (0.95 – 1.35)	0.157	1.11 (0.83 – 1.50)	0.478	0.87 (0.79 – 0.96)	0.006
Heatwave 4-2003, 2^nd ^part	2.33 (1.62 – 3.53)	0.000	1.45 (1.23 – 1.71)	0.000	1.07 (0.79 – 1.44)	0.673	0.99 (0.89 – 1.09)	0.801
Heatwave 4-2003, 3^rd ^part	4.55 (3.51 – 5.90)	0.000	1.75 (1.53 – 2.01)	0.000	0.97 (0.72 – 1.32)	0.862	0.96 (0.88 – 1.05)	0.402

HD = heat disease; RD = respiratory disease; FF = fractures of femur; CD = circulatory disease

^a ^adjusted for day of week

## Discussion

The heat-wave events of summer 2003 have shown that Europe was vulnerable to the effects of heat-waves on human health. A number of concomitant factors contributed to the high excess mortality in some countries, such as the unexpected length and intensity of the heat-wave, the lack of preparedness of health care and social systems for such an event, the lack of intervention plans and the lack of effective technical solutions [18]. Europe, therefore, seems to be a suitable observational ground in order to show the relationship between hospitalization pattern and, on the other hand, intensity or duration or timing of hot spells in summer 2002 and 2003.

In Veneto, 81% of the population lives in cities with less than 100000 inhabitants. A common approach in this field is to carry out time-series analyses of mortality in big cities – eventually pooling city-specific results. However, heat related effects were found in other countries where the majority of population lives in small towns or rural areas [19].

The relations among air pollutant levels, temperature, and mortality have been investigated in several studies [20]. These studies have produced conflicting results, and it is uncertain whether air pollutants are confounders and/or effect modifiers (i.e., a synergistic effect) of the temperature-mortality association [21-25], or whether they have no effect on mortality with temperature [26-28]. Ozone is associated with daily deaths during the summer; a strong positive correlation between ozone and air temperature does exist, which makes the question of disentangling the effects by means of statistical modeling a difficult one [29]. Ozone produces marked lung inflammation in controlled exposure studies [30-32] and could play a major role in the effect of heatwaves on respiratory disease admissions, but it is unlikely to explain the association between duration of heatwaves and the increase of daily hospital admissions for acute renal failure and electrolytic disorders. Therefore, the lack of air pollution data could affect the validity of our study only to a limited extent.

Our finding – that at least four consecutive hot humid days are required to observe a major increase in hospital admissions – is consistent with previous studies on heat-related mortality. In Philadelphia, from 1964 to 1998, every heatwave linked to high mortality lasted at least four days [7]. In St. Louis, among people aged above 64 years, a sharp increase in mortality was observed in 1980 after more than 5 consecutive days of hot weather exceeding 40.6°C of apparent temperature [8]. In the latter study, duration rather than level of apparent temperature increased mortality in elderly during heatwaves [8], thus supporting our findings of table 1. From a physiological standpoint, the finding supports the idea that high mortality and hospitalization occur as a result of accumulated heat load over a longer period of time with little opportunity for respite. These results have important implications upon ongoing efforts to forecast health-related impacts in the heat watch/warning systems, that was first implemented in 1995 in Philadelphia [33]. The first part of an heatwave having only minor effects supports heat health systems where alerts are based on duration of hot humid days.

Many of heat-related diseases may be preventable with adequate warning and an appropriate response to heat emergences, but preventive efforts are complicated by the short time interval that may elapse between high temperatures and health effects. Therefore, prevention programs must be based around rapid identification of high-risk conditions and persons [20]. The effectiveness of the intervention measures must be formally evaluated, and to this purpose our study achieved the following insights: HD and, to a lesser extent, RD are adequate indicators of heat-related hospitalization; the full effect of heatwaves appears after the first 3–4 days have elapsed; days outside heatwaves within the same summer are a suitable reference period allowing to evaluate excesses of hospitalization. The Epidemiological Department of Veneto will continue to collaborate with regional public health authorities in order to communicate the risks of extreme heat and evaluate current heat emergency response plans with emphasis on their ability to predict mortality and morbidity associated with specific climatologic factors.

In examining the timing of heatwaves across the summer months, all the relevant mortality studies report that in a given summer the first heatwave tends to be the most deadly, whereas subsequent events have little impact [4-7]. Unlike this general view, in a year with multiple events, such as 2003, we found that rates of hospital admission for HD and RD equally peaked in the first (early June) and last heatwave (August). These results suggest that any acquired physiologic adaptations or behavioural changes following initial exposure to high ambient temperature failed to avoid hospital admissions in subsequent prolonged heatwaves. Caution should be however exerted in the interpretation of this finding, since it is based on a single heatwave.

Studies on heatwaves have consistently reported an excess of death from cardiovascular disease or cerebro-vascular disease [20], while dehydratation, heat stroke, acute renal insufficiency [1], and respiratory disease [2] were the main causes of hospital admission in old subjects. The latter findings were confirmed by the present study, where circulatory diseases did not show any consistent relationship with heatwave characteristics. The different pattern of hospital admissions and mortality during heatwaves could be explained by the hypothesis that deaths from circulatory diseases occur rapidly in isolated people before they reach a hospital, but could also be due to chance (random variation over time and space in the spectrum of diseases induced by extreme heat), and bias (poor quality of diagnosis on death certificate, or analysis of hospitalization limited to the primary diagnosis). In order to reconcile the above contrasting results, hospital admission and mortality should be concurrently investigated in years with multiple heatwaves [34].

## Conclusion

Mortality during a heatwave, extensively investigated mostly using time-series approach, was found to increase with heat intensity in elderly, early in the summer season, and mainly due to cardiovascular diseases. Little was known about heat-related morbidity.

A different pattern of heat-related disease was shown by hospital discharge records, where acute renal insufficiency, dehydratation, and respiratory disease in elderly were found to increase with duration, rather than intensity, of heatwaves. Hospital admissions during heatwaves peaked equally in early and late summer.

The full effect of heatwaves appears after the first 3–4 days have elapsed, thus supporting the present heat health systems, where alerts are based on duration of hot humid days. Although the finding is based on a single late heatwave, no physiologic adaptations or behavioural changes seem to have occurred in late summer. The effect of heatwaves on selected hospital admissions should be expected not to decrease throughout the whole summer.

The effectiveness of intervention measures aimed at preventing heat-related diseases must be formally evaluated; to this purpose hospital admissions for selected causes may be used as indicator of heat health consequences.

## Competing interests

The author(s) declare that they have no competing interests.

## Authors' contributions

GM contributed to conception and design of the study. UF and GM participated in the analysis and interpretation of data. CV extracted hospital admissions data from the regional archive of discharge records. EF and PS participated in drafting the manuscript. All authors read and approved the final manuscript.

## Pre-publication history

The pre-publication history for this paper can be accessed here:



## References

[B1] Semenza JC, McCullough JE, Flanders WD, McGeehin MA, Lumpkin JR (1999). Excess hospital admissions during the July 1995 heat wave in Chicago. Am J Prev Med.

[B2] Kovats RS, Hajat S, Wilkinson P (2004). Contrasting patterns of mortality and hospital admissions during hot weather and heat waves in Greater London, UK. Occup Environ Med.

[B3] Johnson H, Kovats RS, McGregor G, Stedman J, Gibbs M, Walton H, Cook L, Black E (2005). The impact of the 2003 heat wave on mortality and hospital admissions in England. Health Stat Q.

[B4] Gover M (1938). Mortality during periods of excessive temperature. Public Health Rep.

[B5] Kalkstein LS (1993). Health and climate change. Direct impacts in cities. Lancet.

[B6] Wolfe MI, Kaiser R, Naughton MP, Mirabelli MC, Yoon SS, Hanzlick R, Henderson AK (2001). Heat-related mortality in selected United States cities, summer 1999. Am J Forensic Med Pathol.

[B7] Davis RE, Knappenberger PC, Michaels PJ, Novicoff WM Changing Heatwave Mortality in US Cities. Proceedings of the 16th Conference on Biometeorology and Aerobiology of the American Meteorological Society, Vancouver.

[B8] Smoyer KE (1998). A comparative analysis of heat waves and associated mortality in St. Louis, Missouri – 1980 and 1995. Int J Biometeorol.

[B9] Spolaore P, Brocco S, Fedeli U, Visentin C, Schievano E, Avossa F, Milan G, Toso V, Vanuzzo D, Pilotto L, Pessina AC, Bonita R (2005). Measuring accuracy of discharge diagnoses for a region-wide surveillance of hospitalized strokes. Stroke.

[B10] Lo scompenso cardiaco nel Veneto: validazione e quadro epidemiologico. http://www.ser-veneto.it/attivita/attivita_scompenso.asp.

[B11] Humidex rating. http://www.ccohs.ca/oshanswers/phys_agents/humidex.html.

[B12] What is the humidex?. http://weatheroffice.ec.gc.ca/mainmenu/faq_e.html#weather4b.

[B13] Zauli Sajani S, Garaffoni G, Goldoni CA, Ranzi A, Tibaldi S, Lauriola P (2002). Mortality and bioclimatic discomfort in Emilia-Romagna, Italy. J Epidemiol Community Health.

[B14] Bouchama A, Knochel JP (2002). Heat stroke. N Engl J Med.

[B15] Conti S, Meli P, Minelli G, Solimini R, Toccaceli V, Vichi M, Beltrano C, Perini L (2005). Epidemiologic study of mortality during the Summer 2003 heat wave in Italy. Environ Res.

[B16] Rabe-Hesketh S, Everitt B (2004). A handbook of statistical analyses using Stata.

[B17] Clayton D, Hills M (1993). Statistical models in epidemiology.

[B18] Heatwaves: risks and responses. http://www.euro.who.int/document/E82629.pdf.

[B19] Kysely J (2004). Mortality and displaced mortality during heat waves in the Czech Republic. Int J Biometeorol.

[B20] Basu R, Samet JM (2002). Relation between elevated ambient temperature and mortality: a review of the epidemiologic evidence. Epidemiol Rev.

[B21] Shumway RH, Azari AS, Pawitan Y (1988). Modeling mortality fluctuations in Los Angeles as functions of pollution and weather effects. Environ Res.

[B22] Sartor F, Snacken R, Demuth C, Walckiers D (1994). Temperature, ambient ozone levels, and mortality during summer in Belgium. Environ Res.

[B23] Sunyer J, Castellsague J, Saez M, Tobias A, Anto JM (1996). Air pollution and mortality in Barcelona. J Epidemiol Community Health.

[B24] Touloumi G, Samoli E, Katsouyanni K (1996). Daily mortality and "winter type" air pollution in Athens, Greece – a time series analysis within the APHEA Project. J Epidemiol Community Health.

[B25] Katsouyanni K, Touloumi G, Spix C, Schwartz J, Balducci F, Medina S, Rossi G, Wojtyniak B, Sunyer J, Bacharova L, Schouten JP, Ponka A, Anderson HR (1997). Short-term effects of ambient sulphur dioxide and particulate matter on mortality in 12 European cities: results from time series data from the APHEA project. Air Pollution and Health: a European Approach. BMJ.

[B26] Driscoll DM (1971). The relationship between weather and mortality in ten major metropolitan areas in the United States, 1962–1965. Int J Biometeorol.

[B27] Kalkstein LS (1991). A new approach to evaluate the impact of climate on human mortality. Environ Health Perspect.

[B28] Samet J, Zeger S, Kelsall J, Xu J, Kalkstein L (1998). Does weather confound or modify the association of particulate air pollution with mortality? An analysis of the Philadelphia data, 1973–1980. Environ Res.

[B29] Schwartz J (2005). How sensitive is the association between ozone and daily deaths to control for temperature?. Am J Respir Crit Care Med.

[B30] Devlin RB, McDonnell WF, Mann R, Becker S, House DE, Schreinemachers D, Koren HS (1991). Exposure of humans to ambient levels of ozone for 6.6 hours causes cellular and biochemical changes in the lung. Am J Respir Cell Mol Biol.

[B31] Kinney PL, Ware JH, Spengler JD, Dockery DW, Speizer FE, Ferris BG (1989). Short-term pulmonary function change in association with ozone levels. Am Rev Respir Dis.

[B32] Balmes JR, Chen LL, Scannell C, Tager I, Christian D, Hearne PQ, Kelly T, Aris RM (1996). Ozone-induced decrements in FEV1 and FVC do not correlate with measures of inflammation. Am J Respir Crit Care Med.

[B33] Kalkstein LS, Greene JS (1997). An evaluation of climate/mortality relationships in large U.S. cities and the possible impacts of a climate change. Environ Health Perspect.

[B34] Mastrangelo G, Hajat S, Fadda E, Buja A, Fedeli U, Spolaore P (2006). Contrasting patterns of hospital admissions and mortality during heat waves: are deaths from circulatory disease a real excess or an artifact?. Med Hypotheses.

